# The use of the Multidimensional Condom Attitude Scale in Chinese young adults

**DOI:** 10.1186/s12955-020-01577-9

**Published:** 2020-10-08

**Authors:** Edmond Pui Hang Choi, Daniel Yee Tak Fong, Janet Yuen Ha Wong

**Affiliations:** School of Nursing, The University of Hong Kong, Hong Kong, 4/F, William M.W. Mong Block, 21 Sassoon Road, Pok Fu Lam, Hong Kong

**Keywords:** Condoms, Reproductive health, Sexual behavior, Sexual health, Sexually transmitted diseases

## Abstract

**Background:**

Attitude towards condom use is an important predictor of consistent condom use. However, this topic is an understudied area in Chinese populations, and no validated Chinese instrument is available to capture condom attitude. To fill this research gap, the present study aimed to evaluate the psychometric properties of the University of California, Los Angeles (UCLA) Multidimensional Condom Attitudes Scale (MCAS) and assessed the attitudes towards condom use amongst Chinese adults aged 18–29 years old.

**Methods:**

In this cross-sectional study, a total of 500 people aged 18–29 years old were randomly recruited in Hong Kong. The primary outcome was the attitude towards condom use as measured by the UCLA MCAS. Factor structure, internal construct validity, known-group validity and internal consistency were assessed.

**Results:**

Instead of the five-factor structure designed by the original developers of the MCAS questionnaire, this study proposed a novel six-factor scale: (1) Reliability and Effectiveness, (2) Excitement, (3) Displeasure, (4) Identity Stigma, (5) Embarrassment about Negotiation and (6) Embarrassment about Purchase. The internal construct validity and reliability of the new scale were high. The revised MCAS could differentiate between subgroups, including gender, sexual orientation and sexual experience. In terms of attitudes, over 40% of the participants believed that condoms are not reliable, though the vast majority of the sample did not perceive any stigma related to condom use. In addition, more than half (55.4%) of the respondents felt embarrassed to be seen when buying condoms while a quarter (25.8%) felt uncomfortable buying condoms at all.

**Conclusions:**

Overall, the psychometric analysis found that attitude to condom use is culturally specific. The study also highlighted the need for more public health campaigns and interventions to help people cope with the embarrassment of purchasing condoms.

## Background

Sexually transmitted infections (STIs) remain a severe global health issue. China, including Hong Kong, is experiencing a rapid increase in HIV/AIDS [1, 2]. A population-based study of 18–26 year olds in Hong Kong found that chlamydia is relatively common in sexually active women (5.8%) and men (4.8%) [3]. The increasing popularity of smartphone online dating and a growing openness towards premarital sex and homosexual relationships have resulted in an increased prevalence of STIs [4–7]. It should also be noted that Hong Kong is an important global city with a population of 7.3 million people [3], and the number of visitors to the region continues to rise. A systematic meta-analysis recently reported that the prevalence of travel-associated casual sex (tourists having sex with local people) could be as high as 20%, with 49.4% of these cases involving unprotected intercourse [8]. This phenomenon could be a contributing factor to the increase in STIs in the local community. To diminish the negative impact of STIs, their primary prevention is very important, especially amongst high-risk groups such as men who have sex with men, young people and sex workers.

An essential method of reducing the spread of STIs is consistent condom use. Meta-analyses have reported that consistent condom use can reduce the risk of HIV infection by 80–95% [9, 10]. Condom use is likewise associated with statistically significant protection of men and women against several types of STIs, including chlamydia, gonorrhoea, herpes simplex virus type 2 and syphilis [11]. Although analyses have shown that different interventions, including behavioural [12] and technology-based [13] approaches, can increase condom usage, inconsistent condom use is still found in different Chinese populations [5, 14]. Moreover, findings from a series of behavioural surveillance surveys from 1998 to 2015 showed no evident improvement in consistent condom use in Hong Kong [15]. Hence, there is an urgent need to further understand the barriers and facilitators of condom use to diminish the spread of STIs.

Understanding the predictors of condom use is key to developing effective interventions and community programmes to increase condom use. Attitude is an important factor in nearly all health behaviour models [16]; specifically, existing evidence suggests that attitude towards using condoms is a strong predictor of their use [17, 18]. Moreover, interventions that do not address attitude often fail to improve safe sexual behaviours [19]. Accordingly, understanding and enhancing a positive attitude towards the use of condoms are of paramount importance to achieve consistent condom use. These will, in turn, help reduce the spread of STIs.

### Need for the present study

Despite the importance of understanding the attitude towards condom use, this area is understudied in Chinese populations, including Hong Kong. Study findings obtained in other populations are not necessarily generalisable to Chinese populations because sexual health-related attitude, including attitude towards condom use, is not universal but is influenced instead by the social and cultural norms in each context [20, 21]. A classic example is the opposition of the Roman Catholic Church to the use of artificial contraception, including condoms [22]. Besides, sexual health attitudes are not static and can change generationally [23]. To further enhance sexual health, diminish the adverse impact of non-condom use amongst Chinese populations and develop tailored intervention for specific populations [24], up-to-date information about attitudes towards condom use amongst Chinese population is therefore required.

The University of California, Los Angeles (UCLA) Multidimensional Condom Attitudes Scale (MCAS) is a questionnaire developed to assess the attitude towards condom use [25]. The questionnaire was originally developed for use amongst college students in the United States [25] and subsequently validated in different populations, such as low-acculturated Hispanic women [26] and Black South African university students [27]. However, the psychometric properties of the UCLA MCAS have not been validated in Chinese populations. Therefore, to address these research gaps and provide additional information about attitudes towards condom use in Chinese populations, the present study aimed to evaluate the psychometric performance of the MCAS in young Chinese adults as well as their attitudes to condom use.

## Methods

### Study design

This was a cross-sectional study conducted in Hong Kong.

### Subjects

Mobile phone numbers in Hong Kong were randomly sampled by an independent research organisation, and invitations to participate in the survey were sent by text message to the sampled numbers. Mobile sampling instead of landline sampling was adopted in this study because of the rapidly increasing number of households without landline and the growing concerns about the representativeness of samples selected using landline random digit dialling sampling frames [28]. A person eligible to join the study should (1) be aged between 18 and 29 years old, (2) be able to read Chinese, (3) have a mobile phone and (4) have Internet access. People were excluded if they were not willing to join the study.

### Study instruments

The UCLA MCAS [25] was used in the present study for the following reasons. First, the MCAS was developed amongst young people with a mean age of 19, similar to the population in the present study (the mean age of study participants was 21.2). Second, the development of the MCAS involved people both with and without sexual experience [25], meaning the instrument is not limited to sexually active individuals. Therefore, this instrument was suitable to be used in the current study sample, which was composed of people with and without sexual experience. Third, the ability of MCAS to assess multiple components of attitude is important because attitude towards condom use is multidimensional in nature [18]. The original MCAS is a 25-item instrument arranged across five factors: (1) reliability and effectiveness of condoms, (2) sexual pleasure associated with condom use, (3) stigma attached to those who use condoms, (4) embarrassment about condom use negotiation with sexual partners and (5) embarrassment about condom purchase [25]. Using the MCAS, participants were asked to respond to a seven-point Likert scale that ranges from *strongly disagree* to *strongly agree*, with higher scores indicating a more positive attitude towards condom use.

Demographic information, including age, gender, sexual orientation and sexual experience, were also collected.

### Data analysis

To carry out psychometric analysis of the MCAS, the factor structure, internal construct validity, known-group validity and reliability of the scale were evaluated. Data were fitted to the five-factor model proposed by the original authors [25] and the revised five-factor model by Starosta et al. [18]. However, the data did not fit either of these models. Consequently, the data were split into training (n = 250) and test (n = 250) sets [29]. The training set was used in an exploratory factor analysis (EFA) using principal axis factoring with promax rotation of factors with eigenvalues of > 1.0. A scree plot of the EFA was also used to determine the number of latent factors. The appropriateness of the factor analysis was examined using Bartlett’s test of sphericity and the Kaiser–Meyer–Olkin (KMO) index of sampling adequacy. Bartlett’s test is a measure of the probability that the initial correlation matrix is an identity matrix; to recommend the suitability of the EFA, the *p* value of Bartlett’s test must be lower than 0.05 and the KMO value must be greater than 0.7 [30]. Individual items with a factor loading of ≥ 0.5 were assigned to that factor. Confirmatory factor analysis (CFA) was then tested using the weighted least squares mean and variance that accounted for the categorical nature of the items. Criteria for an acceptable fit were a root mean square error of approximation (RMSEA) of < 0.06, a comparative fit index (CFI) of ≥ 0.90 and a Tucker–Lewis index (TLI) of ≥ 0.90 [31, 32].

Internal construct validity was evaluated using the corrected item-total correlation. A correlation coefficient of ≥ 0.4 was considered an adequate correlation [33]. To evaluate known-group validity, the domain scores were compared by an independent *t* test between (1) male and female respondents, (2) heterosexual and bi- or homosexual participants and (3) people with and without sexual experience. Cohen’s d effect sizes were also calculated and interpreted as either trivial (< 0.2), small (≥ 0.2 and < 0.5), moderate (≥ 0.5 and < 0.8) or large (≥ 0.8) [34]. The internal consistency of the scale was evaluated by the Cronbach’s alpha coefficient, in which a value of ≥ 0.7 was considered to be adequate internal consistency [33].

### Sample size justification

Based on previous guidelines, a sample size of at least 200 people is needed for EFA [35]. In the present study, a total of 500 participants were included in the analysis. This sample size was enough to conduct both EFA (n = 250) and CFA (n = 250).

## Results

### Participant characteristics

Five hundred participants were recruited and completed the online survey. Their mean age was 21.2 years (standard deviation: 2.88), 60.0% were female, 87.4% were heterosexual and 40.0% received tertiary or higher education. Table 1 presents the demographic information of the study participants.

**Table 1:** Demographic information of study participants

Mean age (SD), n = 500	21.23 (2.88)
Gender	n (%)
Male	200 (40.0%)
Female	300 (60.0%)
Sexual orientation	
Heterosexual	437 (87.4%)
Bisexual/homosexual/ others	63 (12.6%)
Education level	
Secondary or below	242 (48.4%)
Tertiary or higher education	200 (40.0%)
Did not answer	58 (11.6%)
Monthly income	
HKD19,999 or below	364 (72.8%)
HKD20.000 or above	29 (5.8%)
Did not answer	107 (21.4%)
Sexual experience	
Yes	390 (78.0%)
No	110 (22.05)

*SD* standard deviation

### Factor analysis and internal consistency

Bartlett’s test for the significance of the correlation matrix was 2437.79 (*p* value < 0.001), indicating the appropriateness for factor analysis. Sample adequacy was confirmed by a KMO value of 0.74, which exceeded the minimum adequacy of 0.50. Principal axis factoring suggested a six-factor structure, accounting for 50.67% of the variance (12.67% by factor 1 ‘Reliability and Effectiveness’, 12.56% by factor 2 ‘Embarrassment about Purchase’, 11.26% by factor 3 ‘Identity Stigma’, 6.41% by factor 4 ‘Embarrassment about negotiation’, 4.32% by factor 5 ‘Excitement’, and 3.45% by factor 6 ‘Displeasure’. Items 6 and 20 returned factor loadings of < 0.5 and did not, therefore, load on any factors, meaning they were removed. Table 2 shows the results of the EFA.

**Table 2:** The result of exploratory factor analysis

*Reliability and effectiveness*		
1	Condoms are an effective method of birth control	0.824
2	The condom is a highly satisfactory form of contraception	0.880
3	I think condoms are an excellent means of contraception	0.695
4^a^	Condoms are unreliable	0.692
5^a^	Condoms do not offer reliable protection	0.547
Variance		12.67%
*Excitement*		
9	Condoms are a lot of fun	0.750
10^a^	The use of a condom is an interruption of foreplay	0.796
6^b^	The use of condoms can make sex more stimulating	0.382
Variance		4.32%
*Displeasure*		
7^a^	Condoms ruin the sex act	0.651
8^a^	Condoms are uncomfortable for both partners	0.643
Variance		3.45%
*Identity stigma*		
11^a^	Men who suggest using a condom are really boring	0.660
12^a^	If a couple is about to have sex and the man suggests using a condom, it is less likely that they will have sex	0.817
13^a^	Women think men who use condoms are jerks	0.720
14^a^	A woman who suggests using a condom does not trust her partner	0.646
15^a^	People who suggest condom use are a little bit geeky	0.642
Variance		11.26%
*Embarrassment about negotiation*		
16^a^	When I suggest using a condom I am almost always embarrassed	0.606
17^a^	It is really hard to bring up the issue of using condoms to my partner	0.737
18	It is easy to suggest to my partner that we use a condom	0.701
19	I’m comfortable talking about condom with my partner	0.762
20^ab^	I never know what to say when my partner and I need to talk about condoms or other protection	0.245
Variance		6.41%
*Embarrassment about purchase*		
21^a^	It is very embarrassing to buy condoms	0.764
22^a^	When I need condoms I often dread having to get them	0.788
23	I don’t think that buying condoms is awkward	0.503
24^a^	It would be embarrassing to be seen buying condoms in a store	0.670
25^a^	I always feel really uncomfortable when I buy condoms	0.615
Variance		12.56%

Exploratory factor analysis (EFA) using principal axis factoring with promax rotation of factors with eigenvalues of > 1.0 was used in the analysis

^a^Items are reverse scored. The score of all reversed items were reordered in the EFA so that all items measured in the same direction

^b^Item 6 and 20 were removed because the factor loading was < 0.5

The CFA provided an RMSEA of 0.056, a CFI value of 0.96 and a TLI value of 0.95. These goodness-of-fit indices indicated that the data fit the six-factor model. Table 3 shows the CFA results. The factor structure is shown in Additional file 1: Figure 1. The item-total correlations for overlap were > 0.4 for all items, except for item 23. The results are presented in Table 4.

**Table 3:** The result of confirmatory factor analysis

	Chi-square value of model fit	*DF*	*p* value	CFI	TLI	RMSEA	90% CI
5-Factor model by the original authors	1262.801	265	< 0.01	0.896	0.882	0.087	0.082, 0.092
5-Factor model by Starosta	1109.094	199	< 0.01	0.901	0.885	0.096	0.090, 0.101
6-Factor model	368.104	207	< 0.01	0.956	0.947	0.056	0.046, 0.065

*DF* degree of freedom, *CFI* comparative fit index, *TLI* Tucker–Lewis index, *SRMR* standardised root mean square residual, *RMSEA* root mean square error of approximation, *CI* confidence interval

There were correlations between residuals of 8 pairs of items within a same factor, including item 1 and item 2, item 1 and item 3, item 2 and item 3, item 4 and item 5, item 16 and item 17, item 18 and item 19, item 16 and item 18, item 21 and item 24

**Table 4:** Descriptive statistics, internal construct validity and reliability

		Corrected item-total correlation^a^	Mean score (SD)^a^	Response distribution of individual items^b^							Combined disagree	Combined agree
				Totally disagree	Disagree	Slightly disagree	Neutral	Slight agree	Agree	Totally agree		
*Reliability and effectiveness*			20.76 (4.34)									
1	Condoms are an effective method of birth control	0.71	4.50 (1.16)	*0 (0%)*	36 (7.2%)	64 (12.8%)	109 (21.8%)	203 (40.6%)	80 (16.0%)	8 (1.6%)	100 (20.0%)	291 (58.2%)
2	The condom is a highly satisfactory form of contraception	0.70	4.44 (1.15)	4 (8%)	34 (6.8%)	61 (12.2%)	124 (24.8%)	197 (39.4%)	78 (15.6%)	2 (0.4%)	99 (19.8%)	277 (55.4%)
3	I think condoms are an excellent means of contraception	0.64	4.45 (1.03)	*0 (0%)*	23 (4.6%)	60 (12.0%)	157 (31.4%)	193 (38.6%)	63 (12.6%)	4 (0.8%)	83 (16.6%)	260 (52.0%)
4^c^	Condoms are unreliable	0.63	3.59 (1.14)	4 (0.8%)	35 (7.0%)	39 (7.8%)	195 (39.0%)	133 (26.6%)	91 (18.2%)	3 (0.6%)	78 (15.6%)	227 (45.4%)
5^c^	Condoms do not offer reliable protection	0.55	3.78 (1.06)	3 (0.6%)	41 (8.2%)	48 (9.6%)	204 (40.8%)	158 (31.6%)	45 (9.0%)	1 (0.2%)	92 (18.4%)	204 (40.8%)
Cronbach’s alpha coefficient^a^		0.84										
*Displeasure*			6.43 (1.74)									
7^c^	Condoms ruin the sex act	0.48	3.20 (1.00)	2 (0.4%)	8 (1.6%)	37 (7.4%)	124 (24.8%)	211 (42.2%)	108 (21.6%)	10 (2.0%)	47 (9.4%)	329 (65.8%)
8^c^	Condoms are uncomfortable for both partners	0.48	3.23 (1.02)	2 (0.4%)	15 (3.0%)	29 (5.8%)	129 (25.8%)	206 (41.2%)	113 (22.6%)	6 (1.2%)	46 (9.2%)	325 (65.0%)
Cronbach’s alpha coefficient^a^		0.65										
*Excitement*			7.94 91.65									
9	Condoms are a lot of fun	061	3.96 (0.85)	6 (1.2%)	28 (5.6%)	63 (12.6%)	298 (59.6%)	94 (18.8%)	11 (2.2%)	*0 (0%)*	97 (19.4%)	105(21.0%)
10^c^	The use of a condom is an interruption of foreplay	0.61	3.98 (0.97)	1 (0.2%)	13 (2.6%)	127 (25.4%)	248 (49.6%)	66 (13.2%)	37 (7.4%)	8 (1.6%)	141 (28.2%)	111 (22.2%)
Cronbach’s alpha coefficient^a^		0.76										
*Identity stigma*			26.55 (3.57)									
11^c^	Men who suggest using a condom are really boring	0.58	5.39 (0.94)	29 (5.8%)	232 (46.4%)	171 (34.2%)	48 (9.6%)	12 (2.4%)	8 (1.6%)	*0 (0%)*	432 (86.4%)	20(4.0%)
12^c^	If a couple is about to have sex and the man suggests using a condom, it is less likely that they will have sex	0.67	5.28 (0.95)	22 (4.4%)	213 (42.6%)	181 (36.2%)	60 (12.0%)	17 (3.4%)	7 (1.4%)	*0 (0%)*	416 (83.2%)	24 (4.8%)
13^c^	Women think men who use condoms are jerks	0.61	5.40 (0.90)	32 (6.4%)	227 (45.4%)	164 (32.8%)	64 (12.8%)	12 (2.4%)	*0 (0%)*	1 (0.2%)	423 (84.6%)	13 (2.6%)
14^c^	A woman who suggests using a condom does not trust her partner	0.59	5.21 (1.00)	27 (5.4%)	189 (37.8%)	179 (35.8%)	78 (15.6%)	21 (4.2%)	4 (0.8%)	2 (0.4%)	395 (79.0%)	27 (5.4%)
15^c^	People who suggest condom use are a little bit geeky	0.55	5.27 (0.92)	30 (6.0%)	192 (38.4%)	178 (35.6%)	85 (17.0%)	13 (2.6%)	2 (0.4%)	*0 (0%)*	400 (80.0%)	15 (3.0%)
Cronbach’s alpha coefficient^a^		0.81										
*Embarrassment about negotiation*			18.80 (3.10)									
16^c^	When I suggest using a condom I am almost always embarrassed	0.50	4.59 (1.07)	10 (2.0%)	98 (19.6%)	153 (30.6%)	166 (33.2%)	61 (12.2%)	11 (2.2%)	1 (0.2%)	261 (52.2%)	73 (14.6%)
17^c^	It is really hard to bring up the issue of using condoms to my partner	0.60	4.71 (1.01)	12 (2.4%)	104 (20.8%)	168 (33.6%)	166 (33.2%)	43 (8.6%)	7 (1.4%)	*0 (0%)*	284 (58.6%)	50 (10.0%)
18	It is easy to suggest to my partner that we use a condom	0.53	4.77 (1.05)	*0 (0%)*	16 (3.2%)	26 (5.2%)	164 (32.8%)	153 (30.6%)	133 (26.6%)	8 (1.6%)	42 (8.4%)	294 (58.8%)
19	I’m comfortable talking about condom with my partner	0.54	4.73 (0.98)	1 (0.2%)	11 (2.2%)	21 (4.2%)	178 (35.6%)	174 (34.8%)	106 (21.2%)	9 (1.8%)	33 (6.6%)	289 (57.8%)
Cronbach’s alpha coefficient^a^		0.75										
*Embarrassment about purchase*			18.55 (3.93)									
21^c^	It is very embarrassing to buy condoms	0.68	3.77 (1.08)	2 (0.4%)	23 (4.6%)	88 (17.6%)	202 (40.4%)	121 (24.2%)	56 (11.2%)	8 (1.6%)	113 (22.6%)	185 (37.0%)
22^c^	When I need condoms I often dread having to get them	0.66	3.91 (1.10)	8 (1.6%)	29 (5.8%)	85 (17.0%)	223 (44.6%)	106 (21.2%)	42 (8.4%)	7 (1.4%)	122 (24.4%)	155 (31.0%)
23	I don’t think that buying condoms is awkward	0.38	3.75 (1.05)	1 (0.2%)	61 (12.2%)	123 (24.6%)	228 (45.6%)	51 (10.2%)	34 (6.8%)	2 (0.4%)	185 (37.0%)	87 (17.4%)
24^c^	It would be embarrassing to be seen buying condoms in a store	0.56	3.24 (1.13)	1 (0.2%)	17 (3.4%)	32 (6.4%)	173 (34.6%)	119 (23.8%)	143 (28.6%)	15 (3.0%)	50 (10.0%)	277 (55.4%)
25^c^	I always feel really uncomfortable when I buy condoms	0.57	3.88 (0.96)	1 (0.2%)	24 (4.8%)	73 (14.6%)	273 (54.6%)	76 (15.2%)	51 (10.2%)	2 (0.4%)	98 (19.6%)	129 (25.8%)
Cronbach’s alpha coefficient^a^		0.79										

Item 6 and 20 were removed because of the result of exploratory factor analysis

*SD *Standard deviation

^a^For Cronbach’s alpha coefficients, corrected item-total correlations and the mean scores, all reversed items were recoded. Higher scores indicate a more positive attitude towards condom use.

^b^The response distribution of each individual item before the reserved items were recoded.

^c^Items are reverse scored

### Reliability

The Cronbach’s alpha coefficient was > 0.7 in five of the six subscales while only 0.65 for Displeasure. The results are shown in Table 4.

### Known-group validity

First, the subscale scores of males and females were compared by an independent *t* test. A statistically significant difference was found in the subscales for Reliability and Effectiveness (effect size = 0.28; *p* value < 0.01), Identity Stigma (effect size = 0.22; *p* value = 0.02), Embarrassment about Negotiation (effect size = 0.19; *p* value = 0.03) and Embarrassment about Purchase (effect size = 0.68; *p* value < 0.01). Second, the subscale scores of heterosexual and bi- or homosexual participants were compared, and three subscales showed a statistically significant difference between the two groups. These differences were in Reliability and Effectiveness (effect size = 0.35; *p* value = 0.01), Displeasure (effect size = 0.31; *p* value = 0.02) and Embarrassment about Negotiation (effect size = 0.42; *p* value < 0.01). Third, the subscale scores were compared between participants with and without sexual experience and statistically significant differences were found in the following subscales: Reliability and Effectiveness (effect size = 0.28; *p* value = 0.01), Displeasure (effect size = 0.45; *p* value < 0.01), Embarrassment about Negotiation (effect size = 1.17; *p* value < 0.01), and Embarrassment about Purchase (effect size = 0.40; *p* value < 0.01). The complete results are shown in Table 5.

**Table 5:** Known-group comparison

	Female		Male		Cohen,s D effect Size	*p* value
	n	Mean (SD)	n	Mean (SD)		
Reliability and effectiveness	300	20.27 (4.22)	200	21.50 (4.44)	0.28	< 0.01
Displeasure	300	6.54 (1.61)	200	6.28 (1.92)	0.14	0.12
Excitement	300	7.86 (1.54)	200	8.07 (1.79)	0.13	0.17
Identity stigma	300	26.86 (3.28)	200	26.08 (3.93)	0.22	0.02
Embarrassment about negotiation	300	18.56 (3.02)	200	19.16 (3.21)	0.19	0.03
Embarrassment about purchase	300	17.53 (3.63)	200	20.08 (3.891)	0.68	< 0.01

	Heterosexual		Sexual minorities/others		Cohen’s D effect Size	*p* value
	n	Mean (SD)	n	Mean (SD)		
Reliability and effectiveness	437	20.94 (4.36)	63	19.48 (4.04)	0.35	0.01
Displeasure	437	6.36 (1.71)	63	6.92 (1.92)	0.31	0.02
Excitement	437	7.89 (1.67)	63	8.30 (1.43)	0.26	0.06
Identity stigma	437	26.61 (3.62)	63	26.11 (3.25)	0.10	0.30
Embarrassment about negotiation	437	18.96 (3.09)	63	17.68 (3.02)	0.42	< 0.01
Embarrassment about purchase	437	18.59 (3.99)	63	18.25 (3.61)	0.09	0.52

	With history of sexual experience		Without history of sexual experience		Cohen’s D effect Size	*p* value
	n	Mean (SD)	n	Mean (SD)		
Reliability and effectiveness	390	21.04 (4.15)	110	19.76 (4.86)	0.28	0.01
Displeasure	390	6.26 (1.72)	110	7.04 (1.71)	0.45	< 0.01
Excitement	390	7.92 (1.73)	110	8.01 (1.29)	0.06	0.63
Identity stigma	390	26.66 (3.43)	110	26.15 (4.03)	0.14	0.23
Embarrassment about negotiation	390	19.46 (3.04)	110	16.45 (2.04)	1.17	< 0.01
Embarrassment about purchase	390	18.87 (4.10)	110	17.41 (3.06)	0.40	< 0.01

*SD* standard deviation

Higher scores indicate a more positive attitude towards condom use.

### Attitude towards condom use in young Chinese adults

Slightly more than half of the participants agreed that condoms are an effective (58.2%) and satisfactory (55.4%) method of contraception, while more than 40% thought they are unreliable. A total 65% of the respondents felt that using condoms is uncomfortable or ruins sexual intercourse. Only a few participants perceived stigma attached to the use of condoms (2.6–5.4%). More than half of the participants did not feel embarrassed about negotiating condom use. However, more than half of the participants felt embarrassed to be seen buying condoms, while a quarter felt uncomfortable about buying them at all. The results are shown in Table 4.

## Discussion

This study established the validity and reliability of using the UCLA MCAS with young Chinese adults. However, the initial exploratory factor analysis suggested that the data obtained from the current Chinese population did not fit the structure found in western populations. For example, a previous study on Colombian men and women [36] and low-acculturated Hispanic women [26] replicated the original five-factor structure. Thus, the results suggested that condom use attitude is culturally specific. Related to this, previous studies have found that differences in race and ethnicity influence perceptions about condom use amongst African Americans and Caucasians [37] and the level of unwanted condom use amongst African Americans, Latinos and Caucasians [38]. This result has implications for the findings of the present study. First, interventions to promote positive attitudes towards condom use should be culture-specific [39] because health promotion strategies that are effective in western contexts may not necessarily transfer to Chinese populations. Second, from a psychometric perspective, caution is required when comparing attitude measured by the MCAS across other cultures given the difference in factor structure. Further studies are warranted to investigate any measurement invariance of this particular instrument across different cultures to ensure that the comparison of instrument scores is valid and meaningful [18]. Nevertheless, similar to the validation study on Colombian men and women [36], the present study also found that men have more positive attitudes towards the reliability and effectiveness of condoms and are less embarrassed by condom negotiation and use. This finding implies that further education to enhance female’s attitude towards the effectiveness of condoms and improve their skills in condom use negotiation is needed.

On the basis of the EFA result, items 6 (‘The use of condoms can make sex more stimulating’) and 20 (‘I never know what to say when my partner and I need to talk about condoms or other protection’) were excluded in the current study. This outcome is consistent with that suggested by Starosta et al. [18], who found that these two items functioned differently between the genders and should be removed from the scale. Item 6 was recommended for removal from the scale because it cannot accurately represent female attitudes towards condom use, thus possibly biasing the result obtained from this subscale [18]. Regarding item 20, Starosta et al. [18] pointed out that sexually active heterosexual women must have considerably more favourable attitudes about discussing the use of condoms than do men. Thus, the inclusion of item 20 in the scale might inflate the score in females compared to that in males [18].

Purchasing condoms was found to be far more embarrassing than condom use negotiation in a Chinese population. Specifically, approximately 25% of the participants said they would feel uncomfortable buying condoms, and more than half expressed embarrassment at being seen buying them. By contrast, only 10% of the participants felt it would be difficult to discuss condom use with their partner. Individuals may feel embarrassed about purchasing condoms because it involves a social audience and public behaviour but not about using condoms where the interaction is with just one other person [40]. A study in North America found that purchasing condoms elicits the most embarrassment, whereas using condoms is the least embarrassing [40]. A further study conducted in China, Canada and South Korea similarly found that the embarrassment associated with purchasing condoms exceeds that of using condoms [41]. Note that embarrassment about purchasing condoms impact purchase intent, which in turn significantly reduces the frequency of condom use [41, 42].

This finding has several implications. First, further discussions about this embarrassment and suggestions about how to cope with it are required in sexual health education [41]. Current sexual health education in Hong Kong and initiatives relating to condom use largely focus on the importance of using condoms, the right way to use them and their effectiveness. However, topics related to embarrassment and purchasing behaviours are neglected. Therefore, purchase-related embarrassment and the strategies to cope with it must be considered in sexual health education in order to promote consistent condom use and improve sexual and reproductive health worldwide [41]. Second, the process of obtaining condoms should be improved and made less embarrassing [42]. Undoubtedly, purchasing condoms online is less embarrassing. However, individuals cannot obtain them immediately, and this is particularly relevant to the context of casual sex in which people need condoms instantly. In Hong Kong, condoms are mainly available in convenience stores, supermarkets and pharmacies, but those who are worried about being seen may not wish to buy condoms from such shops. Vending machines for condoms are available but not very common, and so a possible solution is to place more of these machines in public—but relatively private—places, such as public toilets. Fundamentally, more public health campaigns and sexual health education to help cope with embarrassment about purchasing condoms are required [41].

Perhaps surprisingly, the concept of condom reliability and effectiveness was suboptimal in the present study. Only approximately half of the participants agreed that condoms were an effective and satisfactory method of birth control while more than 40% expressed concerns over their reliability. A related research from Singapore found that 42.1% of the adolescents studied had experienced condom slippage and 32.1% had experienced breakage [43]. A negative attitude towards reliability and effectiveness and a fear of upsetting consequences are probable reasons for the high prevalence of inconsistent condom use in the Hong Kong community [5, 44, 45], and these have implications for the findings. First, qualitative studies are required to explore why people think condoms are unreliable and/or ineffective. Second, individuals should be taught how to choose the correct condom size as well as how to use condoms properly to enhance their reliability and effectiveness in birth control and STI prevention.

A moderate gender difference in embarrassment about condom purchase was found. In line with previous studies in China [41] and the United States [46], female participants were more embarrassed about purchasing condoms than do their male counterparts. A potential reason for this greater degree of embarrassment is because ‘a female is purchasing a male method of contraception’ [47]. Other possible reasons in this population are that sex is still largely taboo in Chinese culture and the existence of gender inequality. For example, Chinese people tend to highlight the importance of premarital sexual abstinence for girls rather than for boys [48], a propensity that is likely to make females more embarrassed about buying condoms than do males. In addition to these gender differences, variance in embarrassment about negotiating condom use across sexual orientations was found, with non-heterosexual respondents more embarrassed than their heterosexual counterparts. An explanation for this may be related to psychological and social factors, such as anxiety, loneliness or distress associated with sexual identity [49]. Disparity in the rates of intimate partner violence (IPV) may also lead to this difference. IPV has been shown to be more prevalent in some non-heterosexual groups than in heterosexual populations [50, 51] and suggested to be capable of lowering the efficacy of condom negotiation [52]. Future interventions aimed at enhancing attitude towards condom use should account for the differences in gender and sexual orientation.

Different levels of embarrassment about negotiating condom use were found between participants who had sexual experience and those who did not. Sexually inexperienced people felt more embarrassed about discussing condom use with their partner and buying condoms in the first place. A possible explanation here is that embarrassment about negotiation and purchase is a barrier to initiating sexual activities amongst young adults. However, these associations should be interpreted with caution because causality cannot be inferred from this limited cross-sectional analysis. Thus, further longitudinal studies are needed to explore the temporal relationship between embarrassment and experience.

The present study has some limitations. First, given its cross-sectional nature, the test–retest reliability and responsiveness of the instrument could not be assessed, and further studies are required to establish the psychometric properties. Second, all participants were aged between 18 and 29 years old. Therefore, the findings may not be generalisable to a wider population. That said, the intention of the current study was to study this subgroup specifically because it was previously found that inconsistent condom use is common within it [5]. For example, young adults are more sexually adventurous than older adults [53]. Furthermore, STIs, including chlamydia, are reported to be highly prevalent in this subgroup of the Hong Kong community [3]. Third, given that only attitude towards condom use was measured, other aspects, such as knowledge, self-efficacy and sexual behaviour, should be included in future studies to investigate their relationships with this attitude.

## Conclusion

The validity and reliability of the MCAS were evaluated in young Chinese adults. Instead of the five-factor structure developed in previous studies, a six-factor scale was found, suggesting that condom attitudes are culturally specific. High levels of embarrassment about purchasing condoms and negative attitudes towards their reliability and effectiveness were reported, implying that future health interventions regarding condom use must focus on these inadequately promoted areas.

## Supplementary information

**Additional file 1:** The figure shows the factor structure of the revsied UCLA MCAS.

## Data Availability

The datasets generated and/or analysed during the current study are not publicly available because it contains personal data but are available from the corresponding author on reasonable request.

## Abbreviations

CFA: Confirmatory factor analysis
CFI: Comparative fit index
EFA: Exploratory factor analysis
IPV: Intimate partner violence
KMO: Kaiser–Meyer–Olkin
MCAS: Multidimensional Condom Attitudes Scale
RMSEA: Root mean square error of approximation
STI: Sexually transmitted infections
TLI: Tucker-Lewis index
