# Supplementary material for: The use of the Multidimensional Condom Attitude Scale in Chinese young adults
Source: Health Qual Life Outcomes. 2020 Oct 8;18:331. doi: 10.1186/s12955-020-01577-9 (PMC7545565; doi:10.1186/s12955-020-01577-9)
Supplement: Supplementary file 1 — Additional file 1: The figure shows the factor structure of the revsied UCLA MCAS. [file 12955_2020_1577_MOESM1_ESM.pdf]

Supplementary figure 1

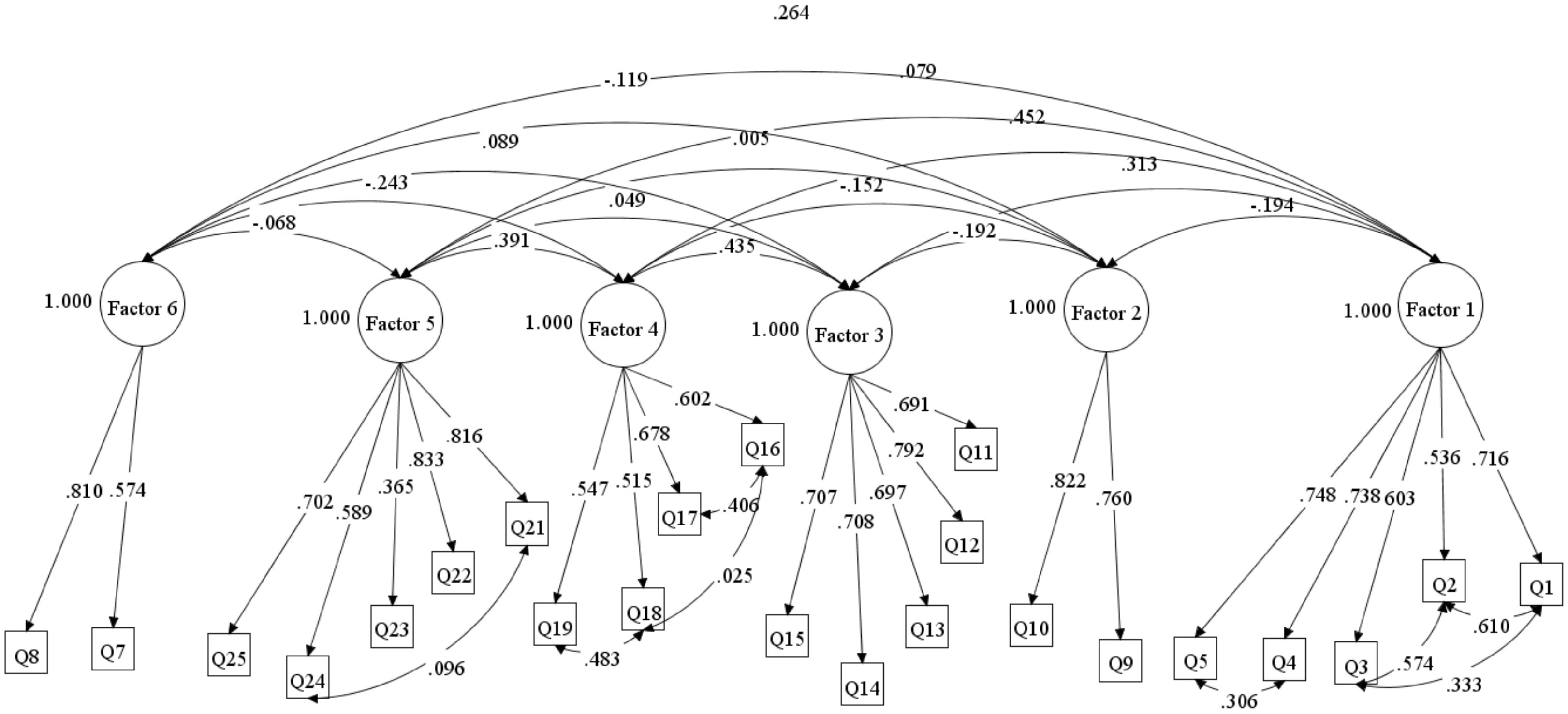

Note:

Factor 1: Reliability and Effectiveness; Factor 2: Excitement; Factor 3: Identity Stigma; Factor 4:Embarrassment about Negotiation; Factor 4: Embarrassment about Purchase; Factor 6: Displeasure
